# Supplementary material for: The dorsal/ventral subdivision of the hindbrain predates the tunicate/vertebrate split
Source: bioRxiv. 2025 Jul 18:2025.07.15.664975. Preprint. [Version 1] doi: 10.1101/2025.07.15.664975 (PMC12338642; doi:10.1101/2025.07.15.664975)
Supplement: Supplement 1 [file media-1.pdf]

Movie S1. pATENs are mechanosensitive. Three *Ciona* larvae responding to being touched at pATENs with a fine probe. The movie plays in real time.

Movie S2. Spiking activity of AMGs. Calcium transients in a *Ciona* larva expressing VGAT>jGCaMP6f. The movie shows 47 seconds of recording at 8.7 frames/second. The movie plays at 5X speed.

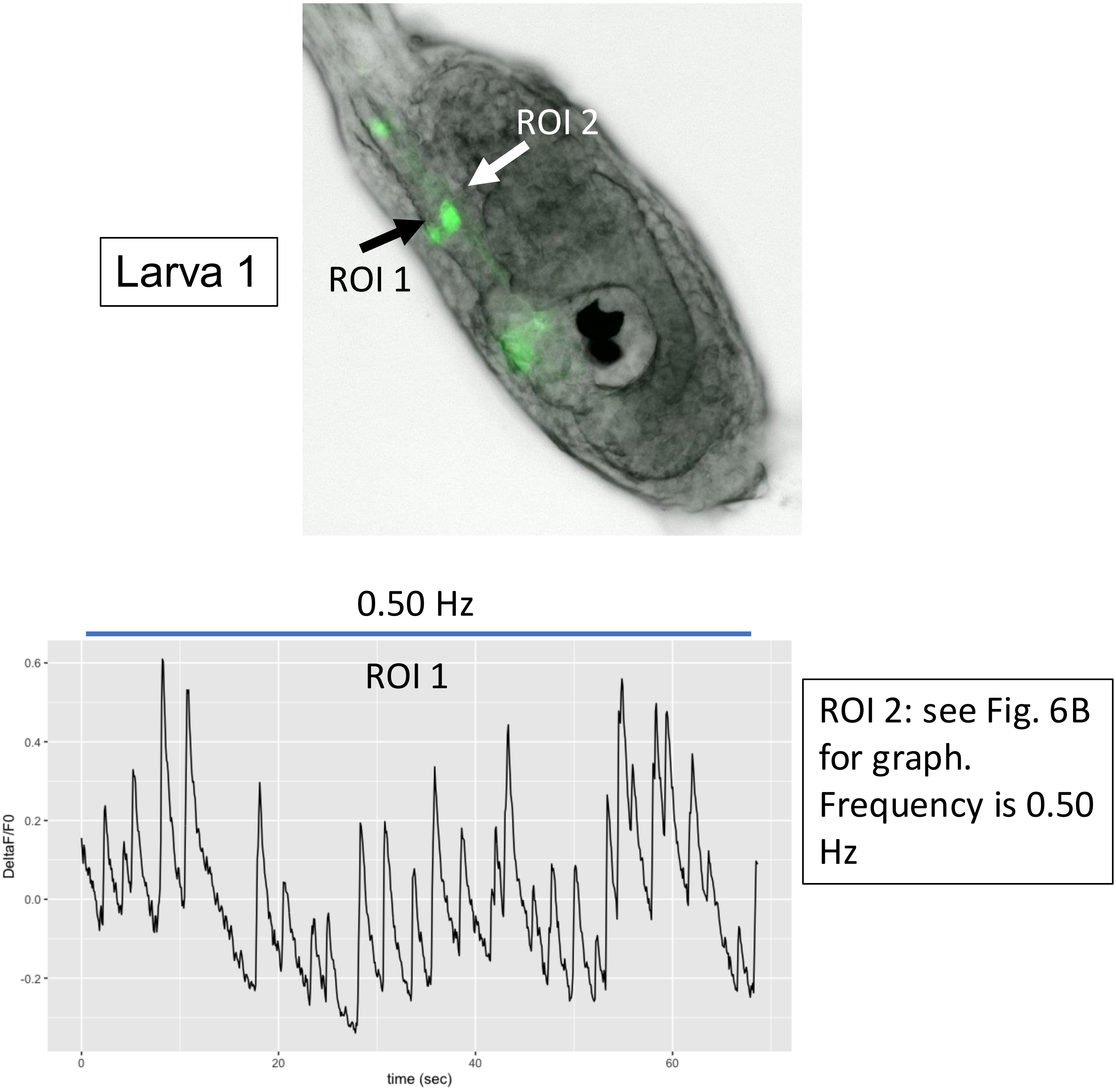

Fig S1. Spontaneous spiking activity in the inhibitory AMG neurons. Data from five larvae transfected with the plasmid VGAT>GCaMP6f. Arrows in left panels indicate the neuron(s) analyzed. Right panels show plots of normalized GCaMP6f fluorescence ($\Delta F/F_0$). Two regions of interest (ROI), corresponding to separate neurons, were analyzed for larva 1. In all other larvae, a single ROI was analyzed (arrows).

Larva 2

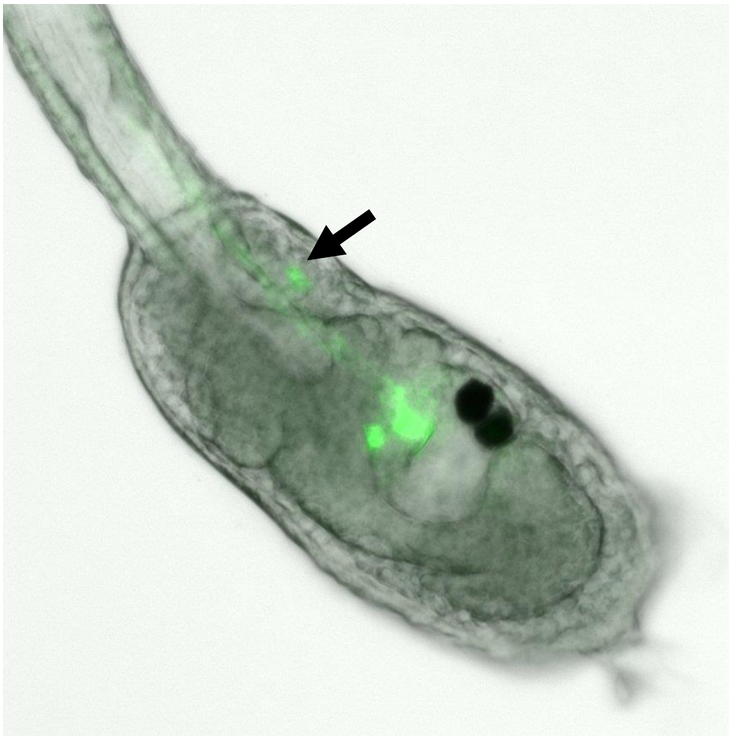

0.39 Hz

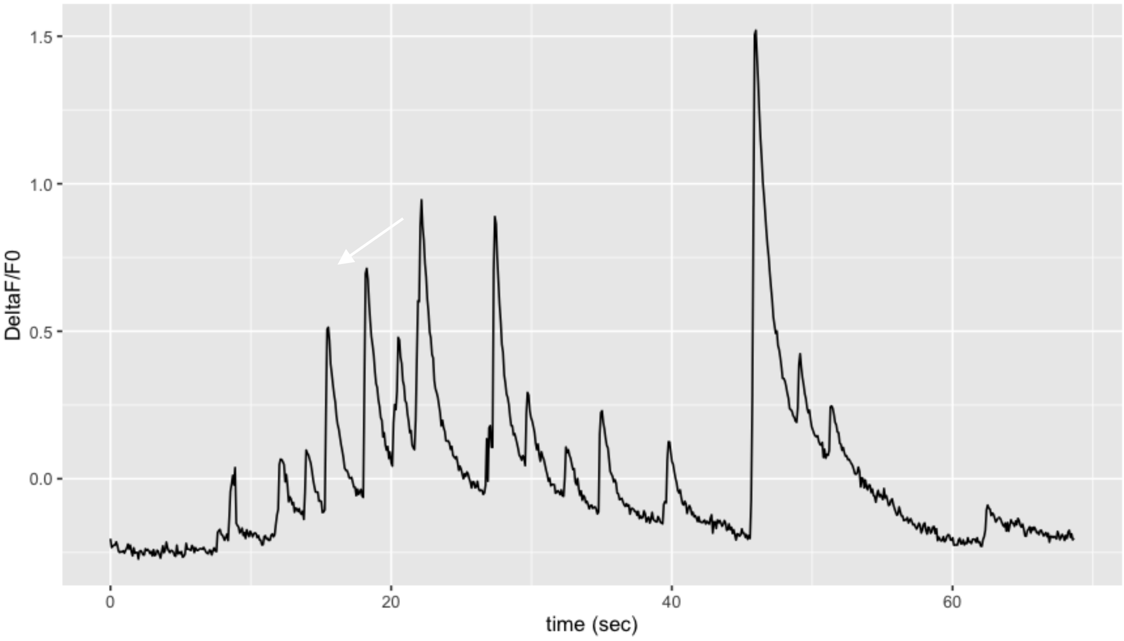

Fig S1 (cont.)

Larva 3

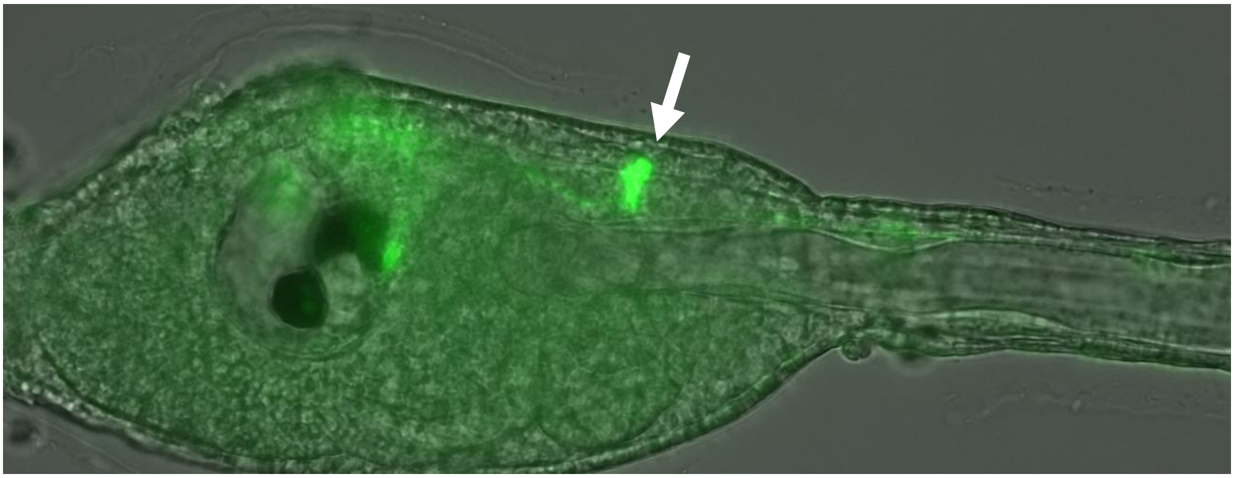

0.53 Hz

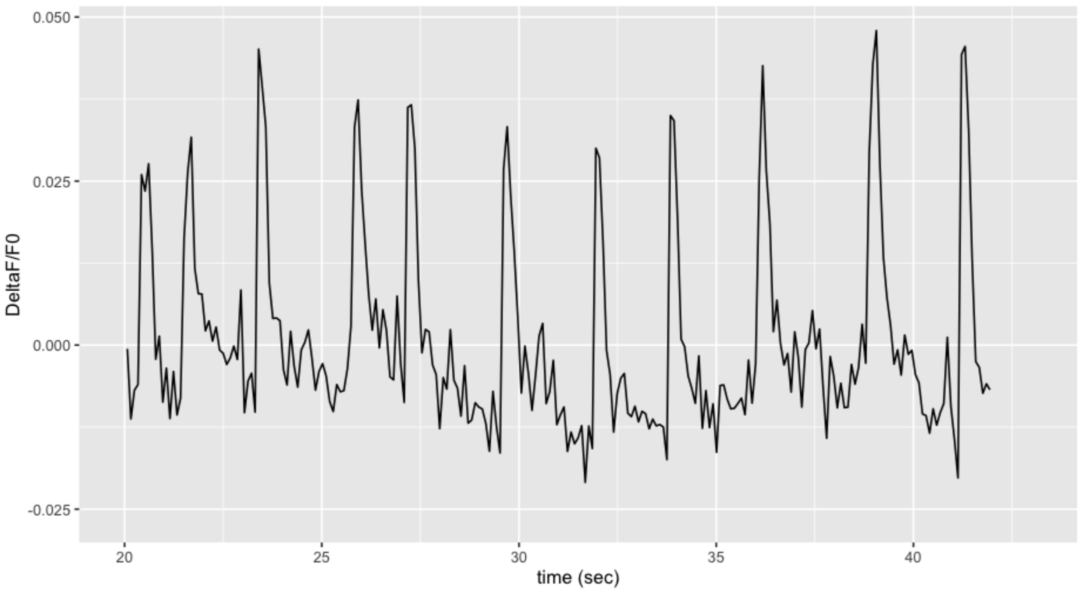

Fig S1 (cont.)

Larva 4

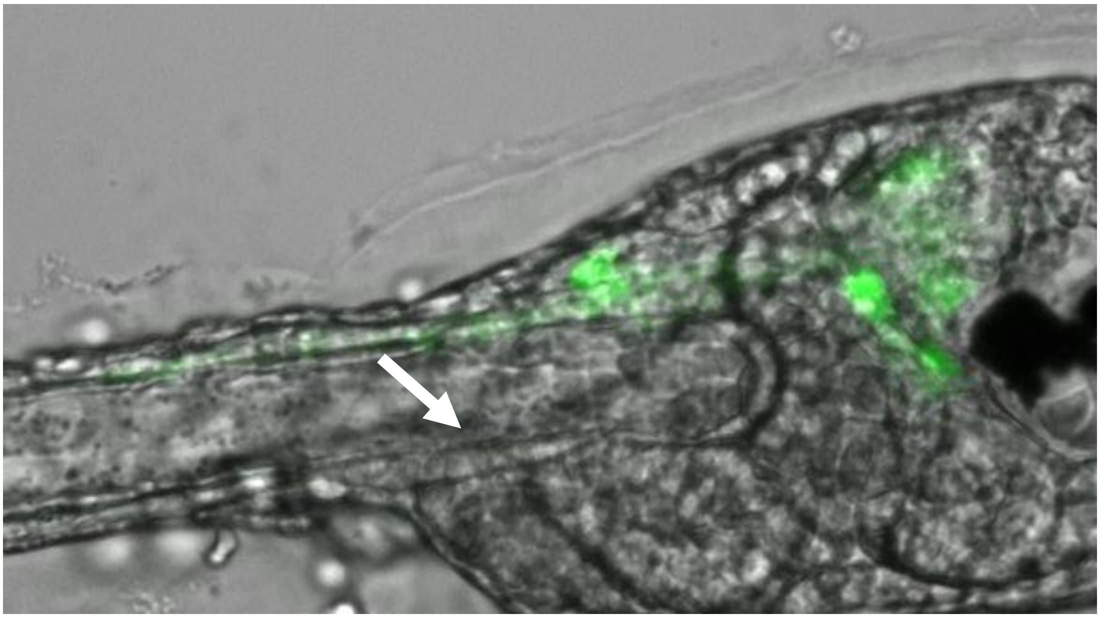

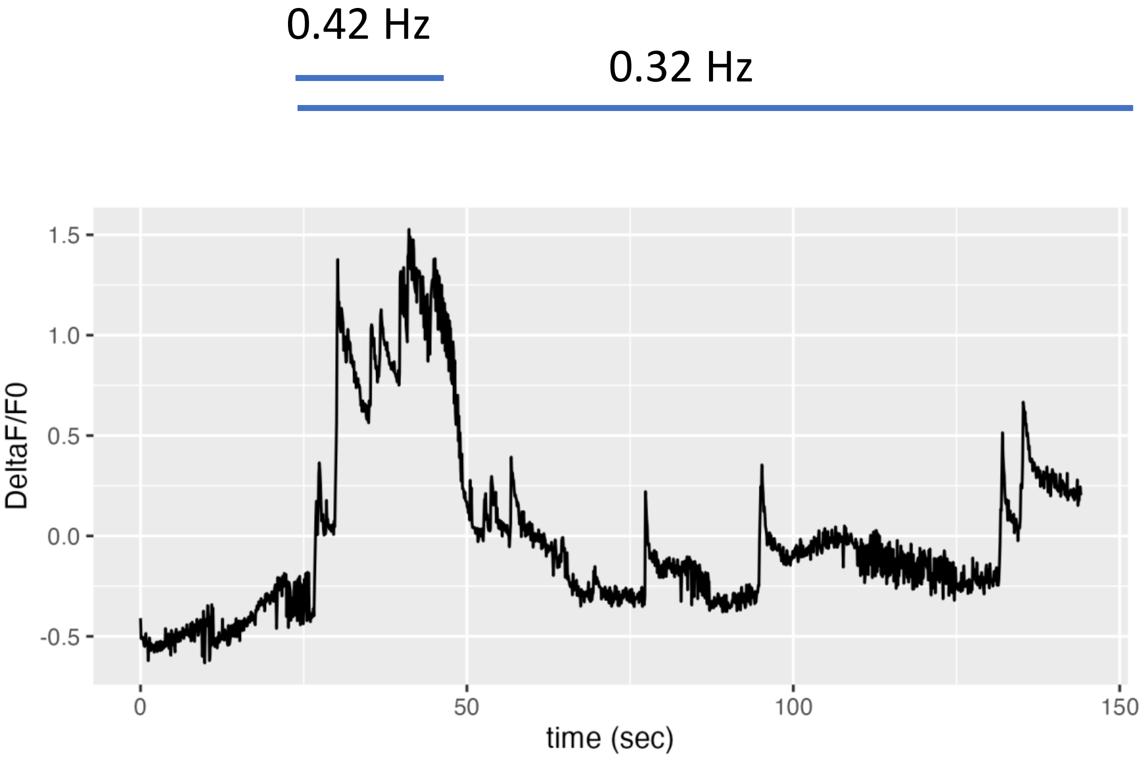

Fig S1 (cont.)

Larva 5

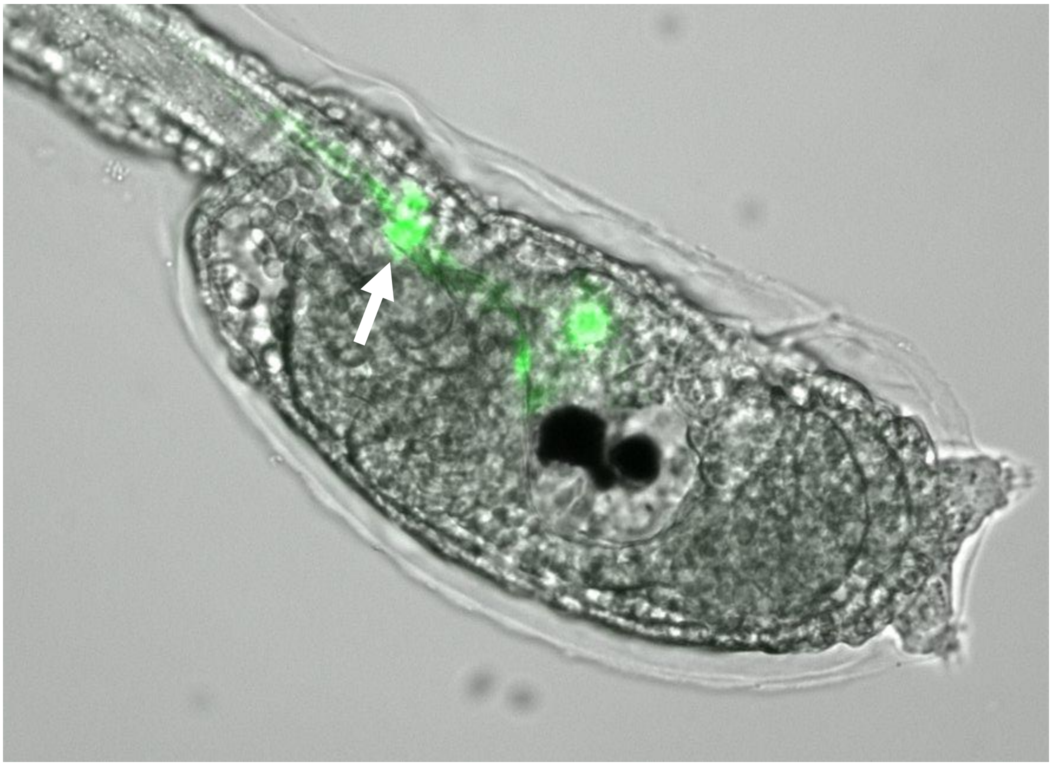

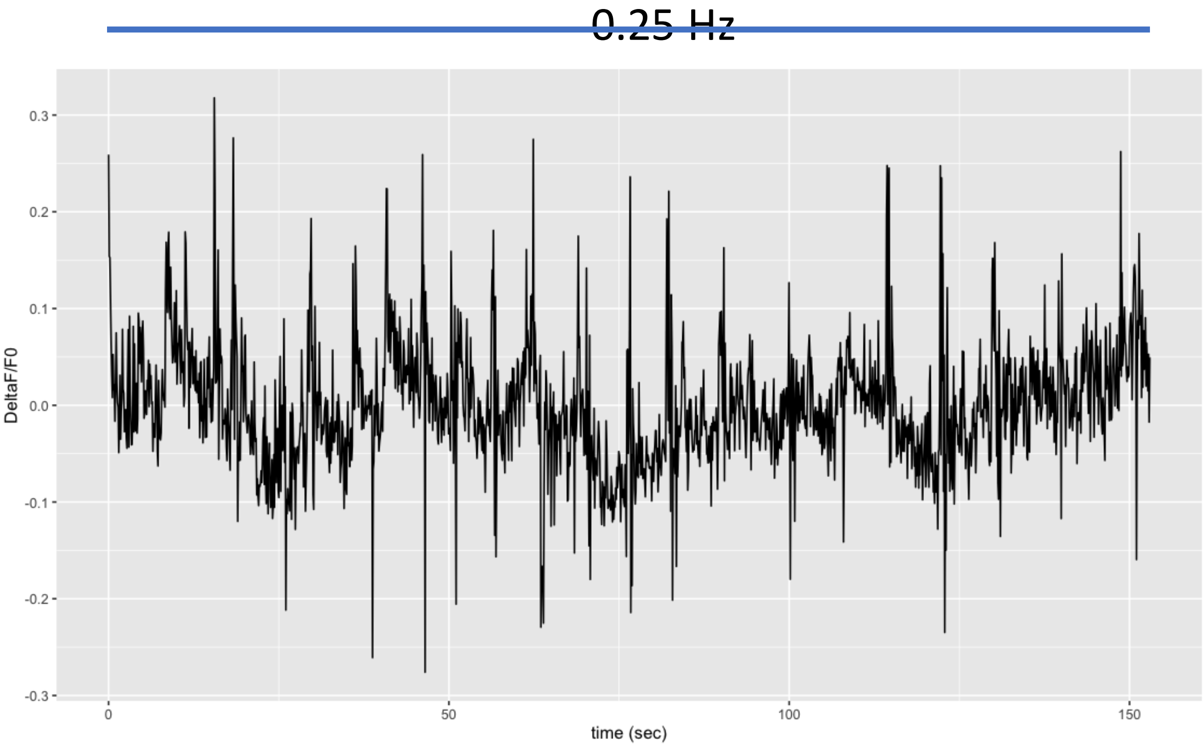

Fig S1 (cont.)

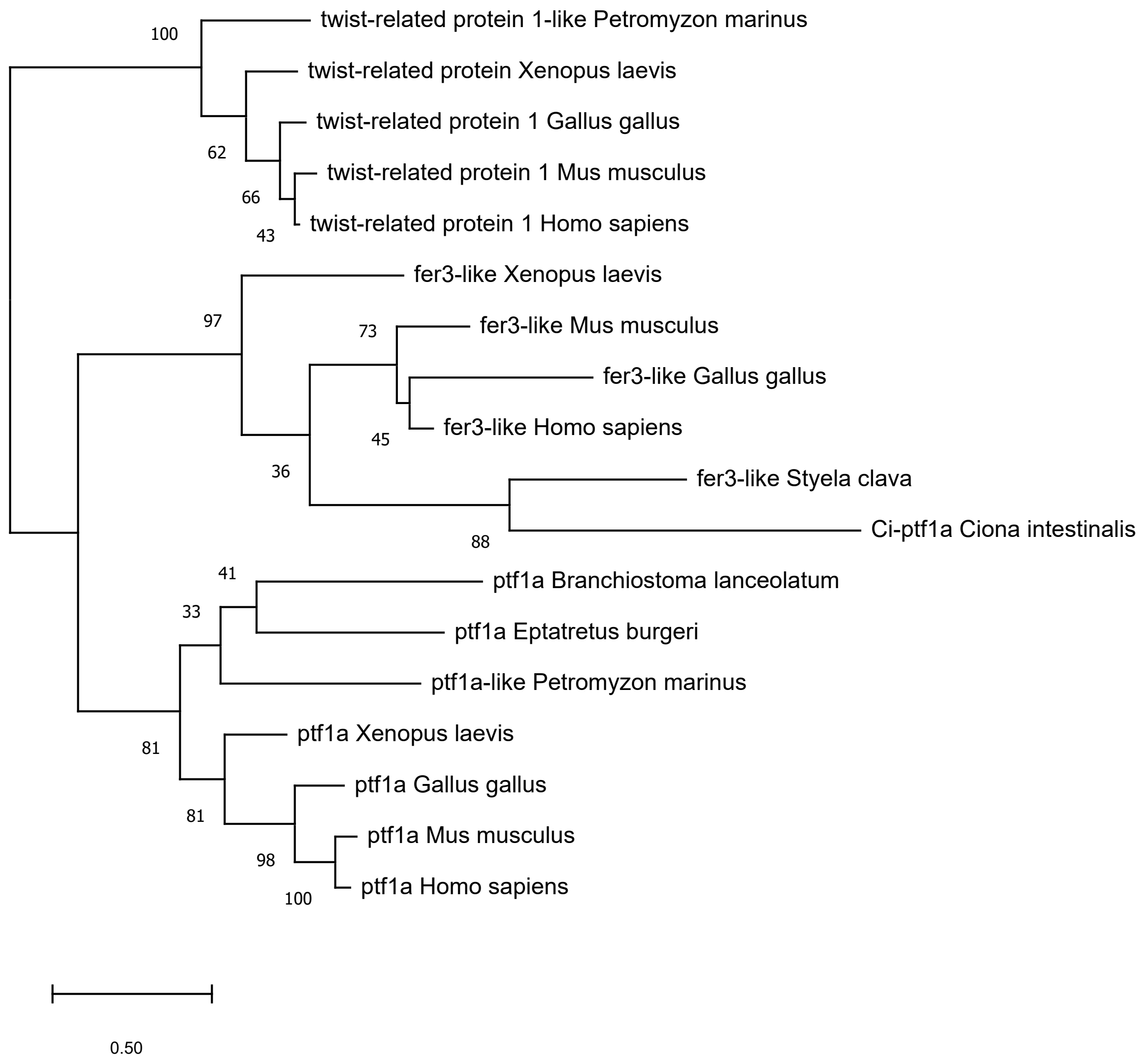

Fig S2. Phylogenic analysis of chordate Ptf1a family members. The putative *Ciona* Ptf1a ortholog (yellow highlight) groups with the fer-like class of transcription factors. Despite the absence of a Ptf1a ortholog in Ciona, an orthologous gene is present in cephalochordates (Branchiostoma lanceolatum; blue highlight).

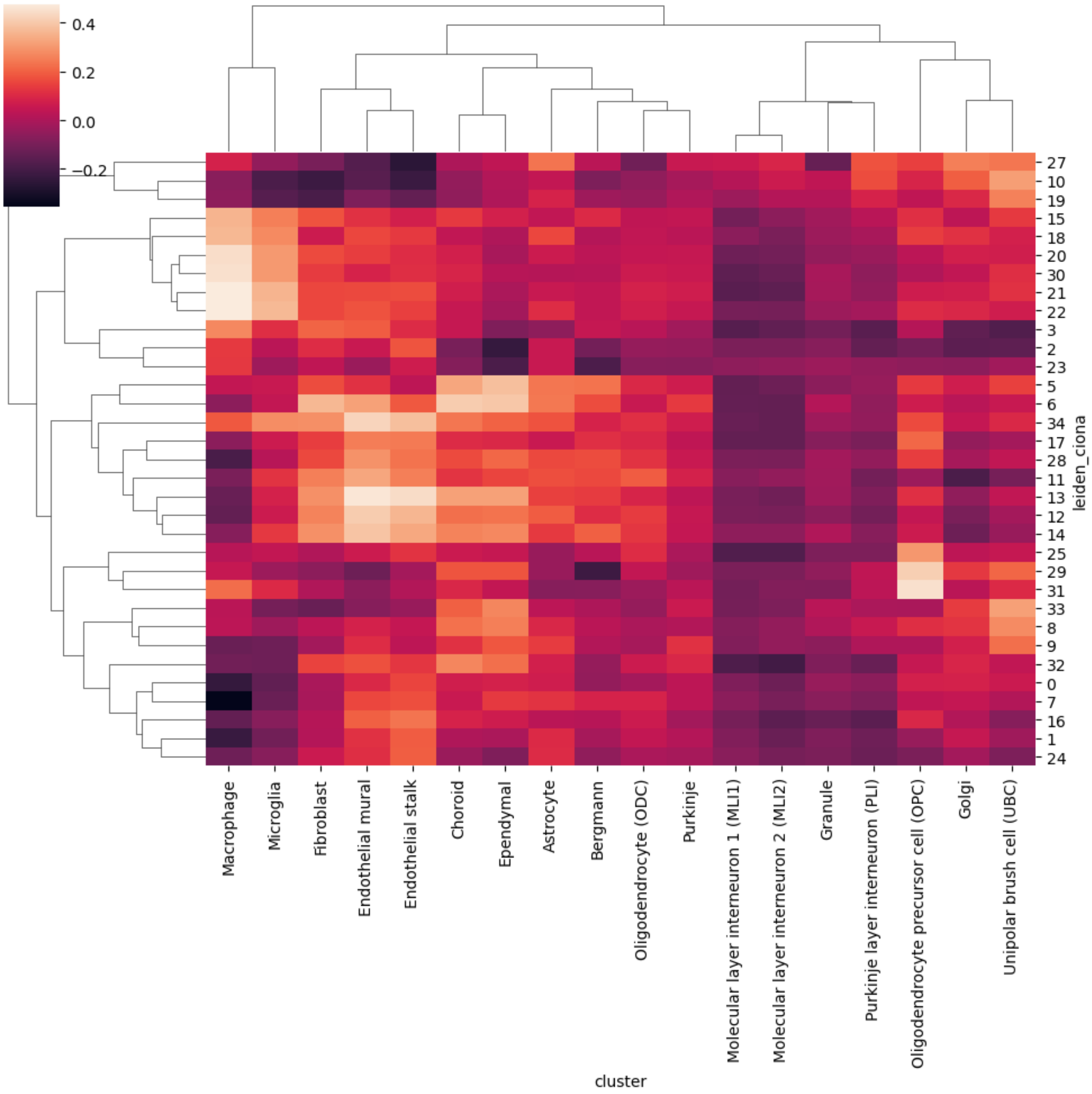

Fig S3. Heat map of mean cosine similarity between annotated mouse cerebellum single cell RNA sequencing clusters and *Ciona* clusters. Several *Ciona* clusters showed considerable expression similarities with mouse cerebellar clusters. However, mouse Purkinje cells cluster does not show an obvious homologous clusters in *Ciona*.

TABLE 1				
Gene ID (KY2021)	lfc_mean	Best Hit Human: uniprot	Best Hit Human: fullname	Notes on expression of vertebrate best hit
KY21.Chr1.2051	7.043459	P78348	Acid-sensing ion channel 1 {ECO:0000303|PubMed:10798398}	Enriched in Purkinje Cells; doi:10.1073/pnas.94.4.1459; doi:10.1038/386173a0
KY21.Chr7.124	6.608743	Q8TBE7	Solute carrier family 35 member G2{ECO:0000312|HGNC:HGNC:28480}	
KY21.Chr1.1244	6.595257	O95672	Endothelin-converting enzyme-like 1	Stongly expressed in Purkinje cells, but not elsewhere in cellebellum; doi: 10.1016/s0306-4522(98)00692-7
KY21.Chr4.865	6.56292	Q02577	Helix-loop-helix protein 2 (HLH-2)	Expressed in developing Purkinje cells; doi: 10.1016/0169-328x(95)00282-w
KY21.Chr2.793	6.445879	Q9H598	Vesicular inhibitory amino acid transporter {ECO:0000303|PubMed:12031963}	Widely expressed in CNS inlcuding Purkinje cells; doi: 10.3389/fncel.2013.00286
KY21.Chr11.1031	6.37433	O95076	Homeobox protein aristaless-like 3	
KY21.Chr2.267	6.364623	Q9HB71	Calcyclin-binding protein	highly enriched in Purkinje cells; DOI: 10.1177/002215540004800903
KY21.Chr1.2142	6.361967	Q99680	G-protein coupled receptor 22	
KY21.Chr11.1275	6.349948	Q9H2C1	LIM/homeobox protein Lhx5	Expressed in Purkinje cells:doi.org/10.1007/s00418-023-02251-z
KY21.Chr4.611	6.336725	NA	NA	
KY21.Chr6.570	6.292621	NA	NA	
KY21.Chr10.677	6.257514	NA	NA	
KY21.Chr11.1122	6.237363	P33032	Melanocortin receptor 5	

table S1. Differentially expressed genes in the inhibitory AMG single-cell transcriptome cluster.

Differential expression (DE) is listed in descending order of $\log_2$ mean fold-change (*mean lfc*). DE genes with a *mean lfc* less than 4.8 and an absolute expression level (*raw normalized mean*) less than 0.2 were excluded. Compiled *mean lfc* for all annotated genes in all clusters is available at https://doi.org/10.5281/zenodo.15320250. Genes with no apparent vertebrate homolog as assessed by BLAST analysis (cutoff E< 0.05) are highlighted in orange. Yellow highlighting corresponds to genes that were used to identify the cluster (VGAT, Lhx1/5 and Otp). Select genes with known expression and/or function in the cerebellum are highlighted in green, along with a reference. Translated *Ciona* gene models [KY2021} can be downloaded from http://ghost.zool.kyoto-u.ac.jp/datas/HT.KY21Gene.protein.2.fasta.zip.

KY21.Chr12.770	6.168701	Q8N475	Follistatin-related protein 5	Highly expressed in cerebellum; doi.org/10.1111/cga.12022
KY21.Chr3.488	6.12561	NA	NA	
KY21.Chr3.1549	6.117251	P21266	Glutathione S-transferase Mu 3	
KY21.Chr14.678	6.102291	Q96EP9	Sodium/bile acid cotransporter 4	
KY21.Chr12.1019	6.091909	Q5TCZ1	SH3 and PX domain-containing protein 2A	
KY21.Chr1.783	6.048831	P28329	Choline O-acetyltransferase	
KY21.Chr11.1143	6.032415	P32297	Neuronal acetylcholine receptor subunit alpha-3	
KY21.Chr2.721	6.024844	Q13394	Putative nucleotidy ltransferase MAB21L1{ECO:0000305}	
KY21.Chr10.378	6.002219	Q05901	Neuronal acetylcholine receptor subunit beta-3	
KY21.Chr3.486	5.991252	NA	NA	
KY21.Chr1.1224	5.978268	P23352	Anosmin-1 {ECO:0000303|PubMed:8832397, ECO:0000312|HGNC:HGNC:6211}	Stimulates outgrowth and branching of developing Purkinje axons; doi:10.1016/j.neuroscience.2008.10.022
KY21.Chr4.240	5.955939	NA	NA	
KY21.Chr4.1089	5.944547	Q9NP94	Zinc transporter ZIP2	
KY21.Chr1.566	5.940061	A7MD48	Serine/arginine repetitive matrix protein 4	
KY21.Chr7.1029	5.936464	Q8TBB6	Solute carrier family 7 member 14	
KY21.Chr12.180	5.926925	P16870	Carboxypeptidase E	
KY21.Chr3.1047	5.895681	P30988	Calcitonin receptor {ECO:0000305}	Enriched in developing cerebellum; https://doi.org/10.1002/cne.10478
KY21.Chr4.864	5.879049	P36383	Gap junction gamma-1 protein	
KY21.Chr10.367	5.876298	P16519	Neuroendocrine convertase 2	

table S1, cont.

KY21.Chr5.104	5.84712	A6NJT0	Homeobox protein unc-4 homolog	
KY21.Chr2.1203	5.827642	P21579	Synaptotagmin-1 {ECO:0000303|PubMed:25705886}	
KY21.Chr14.230	5.807533	Q6UXK2	Immunoglobulin superfamily containing leucine-rich repeat protein 2	
KY21.Chr3.590	5.802678	NA	NA	
KY21.Chr3.20	5.799918	A6NKL6	Transmembrane protein 200C	
KY21.Chr6.500	5.796391	Q5TAB7	Protein ripply2	
KY21.Chr5.1117	5.786946	Q13018	Secretory phospholipase A2 receptor	
KY21.Chr1.1	5.761394	Q92913	Fibroblast growth factor 13 {ECO:0000305}	
KY21.Chr1.1910	5.744116	P24046	Gamma-aminobutyric acid receptor subunit rho-1 {ECO:0000303|PubMed:1849271}	
KY21.Chr9.601	5.730854	Q8N608	Inactive dipeptidyl peptidase 10	
KY21.Chr2.491	5.725424	NA	NA	
KY21.Chr6.24	5.721511	P32297	Neuronal acetylcholine receptor subunit alpha-3	
KY21.Chr3.1580	5.709742	NA	NA	
KY21.Chr10.463	5.709182	O95471	Claudin-7	
KY21.Chr14.449	5.69499	NA	NA	
KY21.Chr2.1365	5.659719	Q92930	Ras-related protein Rab-8B	
KY21.Chr3.9	5.642222	Q96FS4	Signal-induced proliferation-associated protein 1	
KY21.Chr5.81	5.641432	Q9UNE2	Rab effector Noc2	
KY21.Chr7.238	5.633389	P36383	Gap junction gamma-1 protein	
KY21.Chr1.1844	5.633156	Q9Y4B5	Microtubule cross-linking factor 1	
KY21.Chr5.603	5.623641	O14994	Synapsin-3	
KY21.Chr10.618	5.61895	NA	NA	

table S1, cont.

KY21.Chr11.123	5.602503	Q9UI40	Sodium/potassium/calcium exchanger 2	
KY21.Chr14.382	5.602118	O00591	Gamma-aminobutyric acid receptor subunit pi {ECO:0000250|UniProtKB:O09028}	
KY21.Chr1.217	5.600185	Q6IQ22	Ras-related protein Rab-12	
KY21.Chr3.580	5.592871	Q9ULB1	Neurexin-1	
KY21.Chr3.1466	5.569221	P05771	Protein kinase C beta type	
KY21.Chr8.836	5.566675	NA	NA	
KY21.Chr11.1061	5.549929	Q69YW2	Protein stum homolog	
KY21.Chr11.1179	5.541953	NA	NA	
KY21.Chr12.830	5.540315	NA	NA	
KY21.Chr3.886	5.537454	O76038	Secretagogin	
KY21.Chr7.1153	5.529147	NA	NA	
KY21.Chr1.422	5.523847	Q9H4W6	Transcription factor COE3	
KY21.Chr11.193	5.521917	Q6ZNA5	Ferric-chelate reductase 1	
KY21.Chr3.1637	5.5129	Q15907	Ras-related protein Rab-11B	
KY21.Chr3.667	5.507032	Q99622	Protein C10	
KY21.Chr13.97	5.494586	P48544	G protein-activated inward rectifier potassium channel 4	
KY21.Chr1.1592	5.492125	O75325	Leucine-rich repeat neuronal protein 2	
KY21.Chr8.1318	5.479926	A6NHT5	Homeobox protein HMX3	
KY21.Chr8.468	5.474479	Q6PUV4	Complexin-2	
KY21.Chr9.574	5.43528	P28472	Gamma-aminobutyric acid receptor subunit beta-3 {ECO:0000303|PubMed:8382702}	
KY21.Chr1.1459	5.419416	Q9HCR9	Dual 3',5'-cyclic-AMP and -GMP phosphodiesterase 11A	
KY21.Chr9.761	5.414707	Q12879	Glutamate receptor ionotropic, NMDA 2A {ECO:0000305}	
KY21.Chr2.1142	5.404658	Q16555	Dihydropyrimidinase-related protein 2	
KY21.Chr6.262	5.387043	Q9NSD7	Relaxin-3 receptor 1	

table S1, cont.

KY21.Chr6.231	5.369979	O95948	One cut domain family member 2	
KY21.Chr12.916	5.364193	Q13641	Trophoblast glycoprotein	
KY21.Chr2.1366	5.361039	P51787	Potassium voltage-gated channel subfamily KQT member 1 {ECO:0000305}	
KY21.Chr11.360	5.35556	NA	NA	
KY21.Chr1.191	5.333432	P62166	Neuronal calcium sensor 1	
KY21.Chr7.624	5.322394	NA	NA	
KY21.Chr5.876	5.30879	P59768	Guanine nucleotide-binding protein G(I)/G(S)/G(O) subunit gamma-2	
KY21.Chr1.2010	5.303323	Q09470	Potassium voltage-gated channel subfamily A member 1 {ECO:0000305}	
KY21.Chr3.1172	5.27283	Q8NDX2	Vesicular glutamate transporter 3 {ECO:0000303|PubMed:12151341}	
KY21.Chr11.997	5.25814	P50238	Cysteine-rich protein 1	
KY21.Chr2.125	5.257552	O43581	Synaptotagmin-7 {ECO:0000305}	
KY21.Chr12.1064	5.257227	P21452	Substance-K receptor	
KY21.Chr7.1221	5.247487	Q99259	Glutamate decarboxylase 1	
KY21.Chr2.733	5.238503	NA	NA	
KY21.Chr7.252	5.235325	P14416	D(2) dopamine receptor	
KY21.Chr8.957	5.211644	Q9UBP4	Dickkopf-related protein 3	
KY21.Chr10.147	5.201596	O15394	Neural cell adhesion molecule 2	
KY21.Chr7.1003	5.18675	NA	NA	
KY21.Chr1.1311	5.171793	Q9NNX6	CD209 antigen	
KY21.Chr1.1090	5.169981	NA	NA	
KY21.Chr3.140	5.15821	P0C851	Phosphoinositide-interacting protein	
KY21.Chr4.1039	5.15735	Q9H2E6	Semaphorin-6A	
KY21.Chr12.637	5.147467	P0DP23	Calmodulin-1 {ECO:0000312|HGNC:HGNC:1442}	

table S1, cont.

KY21.Chr10.515	5.144052	Q9Y3Q4	Potassium/sodium hyperpolarization-activated cyclic nucleotide-gated channel 4	
KY21.Chr9.379	5.134594	Q13237	cGMP-dependent protein kinase 2	
KY21.Chr11.1113	5.131234	P11229	Muscarinic acetylcholine receptor M1	
KY21.Chr4.922	5.11491	Q9UL51	Potassium/sodium hyperpolarization-activated cyclic nucleotide-gated channel 2	
KY21.Chr9.470	5.113177	Q9UBS5	Gamma-aminobutyric acid type B receptor subunit 1	
KY21.Chr9.1108	5.110392	P37288	Vasopressin V1a receptor	
KY21.Chr11.804	5.109746	Q96SJ8	Tetraspanin-18 {ECO:0000305}	
KY21.Chr8.227	5.109483	Q9BZV3	Interphotoreceptor matrix proteoglycan 2	
KY21.Chr5.741	5.107118	NA	NA	
KY21.Chr5.217	5.096029	P35372	Mu-type opioid receptor	
KY21.Chr2.686	5.08231	Q02153	Guanylate cyclase soluble subunit beta-1 {ECO:0000305}	
KY21.Chr5.906	5.073726	Q9NYY8	FAST kinase domain-containing protein 2, mitochondrial	
KY21.Chr14.175	5.06331	Q9UHG0	Doublecortin domain-containing protein 2	
KY21.Chr14.647	5.053997	Q5XKR4	Homeobox protein orthopedia	Expressed in inhibitory cerebellar cells:doi.org/10.1007/s00418-023-02251-z
KY21.Chr1.863	5.053265	P0DP23	Calmodulin-1 {ECO:0000312|HGNC:HGNC:1442}	
KY21.Chr1.964	5.045961	Q9H4D0	Calsyntenin-2 {ECO:0000303|PubMed:12498782}	

table S1, cont.

KY21.Chr10.1007	5.033205	Q9UMX3	Bcl-2-related ovarian killer protein {ECO:0000303|PubMed:11034351}	
KY21.Chr11.890	5.014723	Q9BTD3	Transmembrane protein 121	
KY21.Chr13.132	5.01279	P48066	Sodium- and chloride-dependent GABA transporter 3	
KY21.Chr13.457	5.012593	Q9UBR4	LIM/homeobox protein Lhx3	
KY21.Chr2.1082	4.981819	P07101	Tyrosine 3-monooxygenase	
KY21.Chr14.837	4.977802	Q9HCJ2	Leucine-rich repeat-containing protein 4C	
KY21.Chr14.109	4.969039	Q96QF0	Rab-3A-interacting protein	
KY21.Chr8.1088	4.967403	P0DPH7	Tubulin alpha-3C chain	
KY21.Chr1.1208	4.963042	P35372	Mu-type opioid receptor	
KY21.Chr13.308	4.950118	NA	NA	
KY21.Chr3.561	4.933967	Q7Z553	MAM domain-containing glycosylphosphatidylinositol anchor protein 2	
KY21.Chr11.400	4.932946	Q9BZE3	BarH-like 1 homeobox protein	
KY21.Chr4.127	4.93143	Q8N2C7	Protein unc-80 homolog	
KY21.Chr9.1072	4.927906	Q9Y6C2	EMILIN-1	
KY21.Chr8.581	4.921591	P49221	Protein-glutamine gamma-glutamyltransferase 4	
KY21.Chr3.263	4.905591	O15344	E3 ubiquitin-protein ligase Midline-1	
KY21.Chr12.1099	4.894771	P0DP23	Calmodulin-1 {ECO:0000312|HGNC:HGNC:1442}	
KY21.Chr4.818	4.874436	Q9ULZ9	Matrix metalloproteinase-17	
KY21.Chr9.1175	4.863217	Q9NQX5	Neural proliferation differentiation and control protein 1	
KY21.Chr9.371	4.862268	Q9NSD5	Sodium- and chloride-dependent GABA transporter 2	

table S1, cont.

KY21.Chr4.553	4.861459	Q16566	Calcium/calmodulin-dependent protein kinase type IV	
KY21.Chr1.2267	4.858945	NA	NA	
KY21.Chr9.941	4.8581	Q86SS6	Synaptotagmin-9	
KY21.Chr1.1533	4.850421	P49795	Regulator of G-protein signaling 19	
KY21.Chr2.768	4.835589	NA	NA	
KY21.Chr6.372	4.81905	NA	NA	
KY21.Chr2.847	4.811907	NA	NA	
KY21.Chr1.291	4.782984	P0DP23	Calmodulin-1 {ECO:0000312|HGNC:HGNC:1442}	
KY21.Chr2.734	4.778576	P42263	Glutamate receptor 3 {ECO:0000305}	
KY21.Chr2.1462	4.746126	Q9NPC2	Potassium channel subfamily K member 9	
KY21.Chr3.1525	4.715206	P0DP23	Calmodulin-1 {ECO:0000312|HGNC:HGNC:1442}	
KY21.Chr1.1655	4.714397	Q8WUI4	Histone deacetylase 7	
KY21.Chr2.441	4.70557	Q9BXA6	Testis-specific serine/threonine-protein kinase 6	
KY21.Chr4.522	4.695408	Q8N441	Fibroblast growth factor receptor-like 1	
KY21.Chr6.434	4.686903	Q92886	Neurogenin-1	
KY21.Chr7.1218	4.677054	Q8IZ57	Neurensin-1	
KY21.Chr13.368	4.673108	Q9GZU5	Nyctalopin	
KY21.Chr10.944	4.662253	P36383	Gap junction gamma-1 protein	
KY21.Chr2.1269	4.597596	Q9UQ13	Leucine-rich repeat protein SHOC-2	
KY21.Chr1.1164	4.589523	NA	NA	
KY21.Chr2.1327	4.580794	NA	NA	
KY21.Chr11.476	4.566051	P48544	G protein-activated inward rectifier potassium channel 4	
KY21.Chr1.2192	4.556142	Q9UI40	Sodium/potassium/calcium exchanger 2	

table S1, cont.

KY21.Chr4.918	4.554426	O15068	Guanine nucleotide exchange factor DBS	
KY21.Chr1.700	4.53225	NA	NA	
KY21.Chr5.850	4.525064	Q96A58	Ras-related and estrogen-regulated growth inhibitor	
KY21.Chr11.329	4.519525	P30874	Somatostatin receptor type 2	
KY21.Chr9.783	4.490294	Q8N568	Serine/threonine-protein kinase DCLK2	
KY21.Chr13.442	4.488406	Q05586	Glutamate receptor ionotropic, NMDA 1 {ECO:0000305}	
KY21.Chr9.1050	4.486921	P26367	Paired box protein Pax-6	
KY21.Chr7.806	4.485088	Q9BRI3	Proton-coupled zinc antiporter SLC30A2 {ECO:0000305|PubMed:22733820}	

table S1, cont.
